# Combining comparative genomics with *de novo *motif discovery to identify human transcription factor DNA-binding motifs

**DOI:** 10.1186/1471-2105-7-S4-S21

**Published:** 2006-12-12

**Authors:** Linyong Mao, W Jim Zheng

**Affiliations:** 1 Department of Biostatistics, Bioinformatics and Epidemiology, and Bioinformatics Core Facility, Hollings Cancer Center, Medical University of South Carolina, 135 Cannon Street, Charleston, SC 29425, USA

## Abstract

**Background:**

As more and more genomes are sequenced, comparative genomics approaches provide a methodology for identifying conserved regulatory elements that may be involved in gene regulations.

**Results:**

We developed a novel method to combine comparative genomics with *de novo *motif discovery to identify human transcription factor binding motifs that are overrepresented and conserved in the upstream regions of a set of co-regulated genes. The method is validated by analyzing a well-characterized muscle specific gene set, and the results showed that our approach performed better than the existing programs in terms of sensitivity and prediction rate.

**Conclusion:**

The newly developed method can be used to extract regulatory signals in co-regulated genes, which can be derived from the microarray clustering analysis.

## Background

Transcription factors (TFs) regulate the expression of genes by interacting with *cis*-regulatory elements in DNA sequences in response to internal and external stimuli. Genomic comparisons have shown that most of these cis-regulatory elements are located in the conserved non-coding region of the genome [1,2]. Over the past decade, many bioinformatics tools have been developed to detect *de novo *DNA motifs bound by TFs that are overrepresented in the promoters of a group of co-regulated genes. Despite the tremendous efforts in algorithm development, discovering human regulatory motifs from a set of co-regulated promoter sequences remains very challenging [3]. In this study, we developed a novel approach to identify human TF DNA-binding motifs for a set of co-regulated genes by combining comparative genomics with *de novo *motif discovery. This approach restricted the motif search in the human promoter regions that are conserved across multiple species.

Most of the current programs combining comparative genomics with *de novo *motif discovery use human-mouse orthologous sequences [4-7] or human-mouse-rat orthologs [8] to obtain the conserved promoter regions. Our approach is the first to use an 8-species (human, chimp, mouse, rat, dog, chicken, fugu and zebrafish) genome comparison to derive the human conserved regions. The method takes the advantage that the ability to detect TF binding sites improves with both the number of comparison species and the evolution distance between species [9,10].

The motif discovery algorithm in our approach is a modification of the original Weeder program [11,12], which is based on the exhaustive oligomer enumeration technique. Current programs that combine comparative genomics with *de novo *motif discovery are based on a greedy search algorithm, Gibbs sampling or expectation maximization techniques [4-8,13,14]. These techniques are all heuristic. Depending on the initial configuration, these heuristic algorithms might be trapped in local maxima [15]. In contrast, due to the exhaustive characteristic of the Weeder algorithm, a single run is sufficient to identify the specified number of most over-represented motifs. In addition, in a recent assessment comparing the performance of various sequence-based motif discovery programs [3], the original Weeder outperformed the other programs, which included the Gibbs sampling and expectation maximization based algorithms, in most measurements. We modified the original Weeder program to incorporate conservation information derived from the comparative genomics. The modified program was implemented in C under Linux.

## Results

### Effects of masking methods

A stringent masking method (SMM) and a window-based masking method (WBMM) were developed to extract conserved upstream regions of human genes from a multiple-species genome alignment. Both methods masked the non-conserved nucleotides and thus reduced the size of sequence space to be searched for regulatory motifs. However, the methods may also eliminate the true TF binding sites as the number of species required to have the same base as human in a multiple alignment column, *t *(see Method), approaches 7. We used the muscle data set to assess the effect of masking methods on the size of searching space and on the percentage of true binding sites retained. The muscle specific gene set was experimentally verified to be regulated by transcription factors Myf, SRF, Mef2, Sp1, Tef and NVL [16]. Fourteen Myf binding sites and 7 Mef2 binding sites determined by experiments were mapped to the upstream sequences of the human genes. For the assessment, an experimentally determined binding site was considered to be retained if a sequence of at least 6 consecutive bases within the binding site was unmasked. Such a binding site can be sampled by a 6-bp motif.

**Figure 1:**
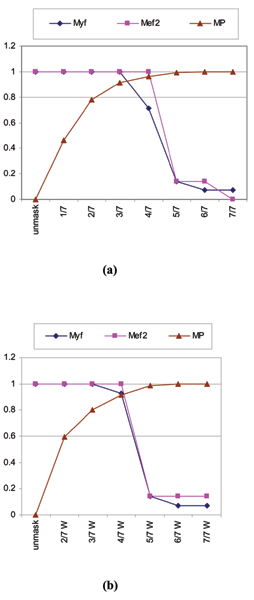
**The effect of (a) SMM and (b) WBMM on the size of sequence space to be searched and on the percentage of true binding sites retained for the muscle genes**. Masking percentage (MP) is defined in Methods. '*t*/7' represents SMM and indicates that a base is retained if the base is in the non-repeat region and conserved in at least *t *of the 7 species (Methods). '*t*/7 W' indicates the corresponding WBMM.

As expected, when more and more stringent conservation criteria were imposed by increasing the *t *value, the number of 6-mers retained in the data set decreased. When *t *was set at 2, 78% of 6-mers in the data set were masked out for SMM and 59% for WBMM; when *t *increased to 4, 96% of 6-mers were masked out for SMM and 91% for WBMM (Figure 1). Since WBMM relaxes conservation criteria imposed by the corresponding SMM, it increased the number of oligomers retained. In contrast to the significant reduction in the overall number of 6-mers, 100% of Mef2 binding sites and at least 70% of Myf binding sites were retained for both SMM and WBMM as long as *t *was set below or equal to 4 (Figure 1).

### Motif discovery for the muscle gene set

Motif discovery results obtained by using different masking parameters were compared (Table 1). When at least one of the seven species was imposed to share the same base with human (*t *= 1) for SMM, only the Myf DNA-binding motif was detected with low sensitivity and low positive prediction rate (PPR). When *t *was set at 3 using SMM, Myf, SRF, Mef2, and NVL DNA-binding motifs were detected with a combined sensitivity rising to 0.56 and PPR 0.42. Among the four detected motifs, the Myf motif predicted 10 out of 14 sites bound by the TF, and the Mef2 motif predicted 6 out of 7 sites bound by the TF. When WBMM was applied and *t *was set at 4, the same four TF DNA binding motifs were detected with combined sensitivity equal to 0.41 and PPR 0.56. Among them, the SRF motif predicted three binding sites of which two were true hits. We also used the 5000-bp upstream sequences (no gap) for which only the repeat regions were masked, and the algorithm detected Myf, SRF and NVL DNA binding motifs (Table 1). However, all three motifs showed low PPR. For example, the PPR for the SRF motif is only 0.016. For comparison, CompareProspector [6] and Toucan [7,14] were also applied to detect DNA binding motifs for the muscle gene set, and the result was included in Table 1. CompareProspector detected four TF DNA binding motifs (Myf, SRF, Mef2 and Tef) with combined sensitivity equal to 0.24 and PPR 0.5. Toucan only detected the Myf and NVL motif, and the combined sensitivity and PPR were 0.09 and 0.18, respectively.

## Discussion

**Figure 2:**
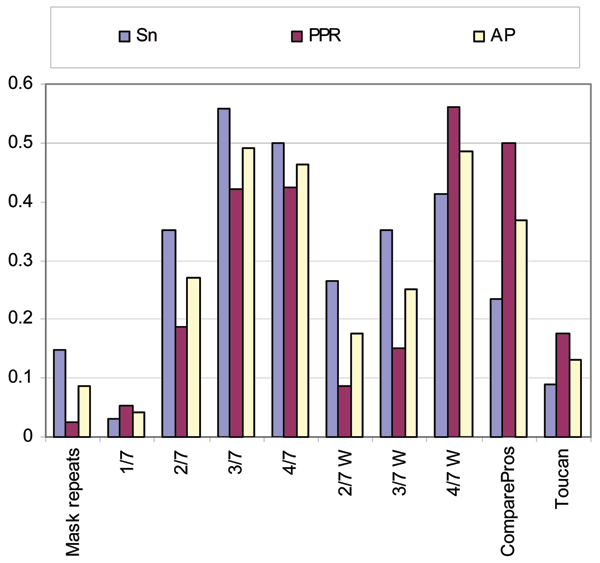
**Comparison of performance of CompareProspector, Toucan and our approach on the muscle genes**. Sn denotes the combined sensitivity of a particular program for the muscle genes, PPR denotes combined positive prediction rate, AP denotes combined average performance (Table 1). 'Mask repeats' represents the 5000 bp upstream sequences (no gap) for which only the repeat regions were masked were used. Refer to Figure 1 for the description of '*t*/7' as well as '*t*/7 W'.

In this study, we combined comparative genomics with *de novo *motif discovery to identify human TF DNA-binding motifs. Based on the 8-species multiple alignments, SMM and WBMM were developed to extract conserved upstream regions of human genes. Using SMM or WBMM with appropriate parameter settings we could substantially reduce the amount of sequence space to be searched for the identification of regulatory motifs while having most of the true binding sites retained (Figure 1). Such properties of the masking method may significantly increase the possibility of finding true binding sites by a motif discovery program, which is evidenced by the motif discovery results for the muscle specific gene set (Figure 2). Compared with the performance of the motifs identified using upstream sequences for which only repeat regions were masked, both the combined sensitivity and PPR for SMM (*t *= 3) as well as for WBMM (*t *= 4) were improved significantly (Figure 2).

Our *de novo *motif discovery algorithm in combination with the masking method outperformed CompareProspector and Toucan according to the motif discovery results for the muscle gene set. Both CompareProspector and our approach using WBMM with *t *set to 4 identified four TF regulatory motifs (Table 1), however, our approach exhibited higher sensitivity and higher prediction rates than CompareProspector (Figure 2). Our approach correctly predicted 14 out of the 34 true binding sites for the muscle genes which substantially exceed the 8 true binding sites identified by CompareProspector. In comparison, Toucan only identified two TF DNA-binding motifs. Both the combined sensitivity and PPR for Toucan are lower than our approach using WBMM with *t *set to 4. Both CompareProspector and Toucan biased motif searches in the human-mouse conserved regions, and they both employ Gibbs sampling technique for the motif discovery. In contrast, our approach requires motifs to be conserved over multiple species and uses exhaustive oligomer enumeration technique to discover motifs. It is likely that both the properties of the masking method and motif discovery technique contribute to the superior performance of our approach.

## Conclusion

Deciphering human regulatory motifs is crucial for understanding the regulatory mechanisms that control gene expression in response to various stimuli. Recent advances in genomics have enabled large scale investigation of gene regulation by microarray technology, which can identify genes with similar expression patterns by clustering analysis. These gene clusters with similar expression patterns are likely to be regulated by common transcription factors. It is feasible to apply the approach developed in this study to analyze the gene clusters for the extraction of regulatory signals.

## Methods

### Extracting conserved upstream regions of human genes

Multiple alignments of 5000 bp sequences upstream of annotated transcription starts of human RefSeq genes to the genomes of the following 7 species, chimp, mouse, rat, dog, chicken, fugu and zebrafish, using the program Multiz [17] were downloaded from UCSC Genome Browser. Two methods were applied to extract conserved upstream regions, respectively. The first one would be referred to as stringent masking method (SMM). The method retained a base in a human gene's upstream sequence if the base was located in the non-repeat region and conserved in at least *t *out of the other 7 species in a multiple-alignment column, where *t *is an integer and specified by a user. The method replaced a base with the letter 'N', otherwise [18]. The second method, referred to as window-based masking method (WBMM), utilized less stringent conservation criteria. It first assigned a score for each base in a human gene's upstream sequence multiple-aligned with the other 7 species. If a base was in the non-repeat region and conserved in at least *t *of the seven species, it was assigned 1; otherwise, a base was assigned 0. The score for a gap inserted into the human gene's upstream sequence for the purpose of multiple-alignment was also assigned 0. Then a window-based value (WBV) for a base in the aligned human upstream sequence was calculated as the summation of scores over a user-specified window size centered at that base. The summation excluded the score of that base. After WBV was calculated, a base was retained if it met either of the following two conditions: (i) the score for the base is 1; (ii) the base is in the non-repeat region and the WBV for the base exceeds a certain threshold specified by a user. Otherwise, the base was masked by 'N'. Eleven bp was chosen as the window size and the threshold for WBV was set at 7 in this study. For both methods, gaps appearing in the multiple-alignment were retained as part of the upstream sequence of a human gene.

### *De novo *motif discovery

The algorithm discovers over-represented motifs in a set of DNA sequences upstream of co-regulated genes using the exhaustive oligomer enumeration technique. The algorithm is a modification of the Weeder program [3,11,12] to cope with the masked bases and gaps in upstream sequences. In the algorithm, an oligomer *m *with length *l *is defined as a sequence of *l *conserved bases. Neither the letter 'N' used to replace a nucleotide nor a gap can appear in *m*. A match to the *m *that allows for *e *mutations is also an *l-*bp oligomer that has at most *e *mismatches with respect to *m*. Let *S = S*_1_*...S*_*k *_be the set of masked upstream sequences, *n*_1_*...n*_*k *_are the number of *l*-bp oligomers in the corresponding sequence, and *N *is given as:



A sequence specific score for the oligomer *m *allowing for *e *mutations is given as [12]:



where *A *represents the set of sequences each of which has at least one match to the *m*, *e*_*i *_(0 ≤ *e*_*i *_≤ e) is the minimal number of mismatches to *m *among all matching oligomers in the *i*^*th *^sequence, *H*_*i*_*(m, e *_*i*_*) *is the number of oligomers with exact *e*_*i *_mismatches with respect to *m *in the *i*^*th *^sequence, and *B (m, e*_*i*_*) *is the background frequency for *m *with *e*_*i *_mutations computed using the genome-wide regulatory regions of the human species [12].

In addition, a general score for *m(e) *was also calculated [12]:



where *Tot(m, e) *is the total number of oligomers matching *m(e) *in all input sequences, *B(m, e) *is the background frequency for *m(e)*.

A final score for *m(e) *is given as:

*Score*(*m*, *e*) = *seq*(*m*, *e*) + *Glo*(*m*, *e*)     (4)

The algorithm used (4) to calculate scores for all oligomers of the same length that occur in the upstream sequences and ranked them based on their scores. Putative TF DNA-binding motifs were selected from the top ranked oligomers. In applying the algorithm to discover DNA-binding motifs for the muscle specific gene set (22 genes), both strands of the genes' upstream sequences were searched. The algorithm enumerated all 6, 8, 10, and 12-bp oligomers in the upstream sequences. The number of mutations allowed is 1 for 6-mer, 2 for 8-mer, 3 for 10-mer and 4 for 12-mer, corresponding to the large mode in the original Weeder program [12]. Only oligomers that were positively scored and for which matching oligomers can be found in more than 50% of the number of input sequences were retained. Among the retained oligomers, the twenty highest scored oligomers, if any, at the lengths of 6, 8, 10 and 12 bp, respectively, were further selected. Among them, only oligomers satisfying both of the following conditions were reported to the user as discovered motifs: (i) An oligomer ranks in the top ten; (ii) An oligomer is both horizontally and vertically redundant, as defined by the Weeder program [12], by comparing the pattern of the oligomer with the other oligomers ranked in the top twenty. Thus, for the muscle gene set, at most 40 motifs were reported. In analyzing the muscle gene set, parameters of the algorithm were set as: -O HS -R 50 -S -M -T 20.

After redundant motifs which also ranked in the top ten were reported, they were used to search for putative TF binding sites in the masked upstream sequences. Assuming one of the reported motifs is an *l*-bp oligomer allowing for *e *mutations, all matching occurrences in the masked upstream sequences were first collected to construct the position specific weight matrix (PSWM). This PSWM was then used to compute a score for each matching occurrence [12]. All the matching occurrences with scores above a certain threshold were output as the putative TF binding sites. In this study, the threshold for a 6-mer is set at 100% (i.e. exact match), and the thresholds for 8- 10- and 12-mers are 85%.

### Masking percentage

SMM or WBMM reduced the size of sequence space to be searched for regulatory motifs. Masking percentage (MP) is introduced to measure such reduction, and it is calculated as:

*MP *= 1 - *N*_6 - *mer*_/(4995 · *k*)     (5)

where *N*_6-*mer *_is calculated using (1) for 6-bp oligomers, 4995 is the number of 6-mers that would be found in an unmasked 5000-bp sequence containing no gaps, and *k *is the number of input sequences.

### Using CompareProspector and Toucan to discover DNA-binding motifs

For comparison, CompareProspector and Toucan were also applied to identify TF regulatory motifs for the muscle genes. CompareProspector is a motif discovery program that extends Gibbs sampling by biasing the search in promoter regions conserved across species [6]. In applying CompareProspector to discover human regulatory motifs, the alignment of human-mouse orthologs were extracted from the 8-species multiple alignment and were used to calculate window (20 bp) percent identity values required by the program. Motif lengths were set at 6, 8, 10 and 12 bp, respectively. For each motif length, the top ten motifs were reported. The parameters for using CompareProspector are '-T 0.8 -Z 0.5 -D 1'. Toucan also used the Gibbs sampling technique, in combination with comparative genomics, to identify DNA-binding motifs [7,14]. The motif(s) identified by Toucan for the muscle genes were taken from Aerts et al [7].

### Evaluating program performance

Sensitivity (Sn) and positive prediction rate (PPR) were computed for a discovered motif. Sn gives the fraction of true binding sites (*i.e*. experimentally established binding sites) that are predicted by a motif, whereas PPR reflects specificity and is calculated as the fraction of sites predicted by a motif that are true binding sites [3]. The mean of Sn and PPR for a discovered motif is defined as average performance (AP) of the motif. In this study, a motif predicted site is considered to be a true hit if it overlaps with a true binding site by at least 5 bp.

## Authors' contributions

Linyong Mao carried out the algorithm development and data analysis, and helped to draft the manuscript. WJZ was the principal investigator, conceived of the project and guided its development. All authors read and approved the final manuscript.

## Figures and Tables

**Table 1:** Comparison of motifs identified by different programs for the muscle genes ^1,2,3,4,5^.

	**Myf**	**SRF**	**Mef2**	**Tef**	**NVL**	**Combined**
masking repeats ^6^	GGGACATG 14/2/68	TCAGCCCT 4/1/63	N	N	ATCAGCCC 4/2/60	34/5/191
1/7	AGGGGGCATG 14/1/19	N	N	N	N	34/1/19
2/7	GACAGCTG 14/9/41	ACAAGG 4/1/5	AAATAGCCCC 7/1/4	GACATCTGGC 4/1/14	N	34/12/64
3/7	CAGCTGTT 14/10/19	CCTTATTTGG 4/2/12	GCTAAAAATAGC 7/6/12	N	CATACAAGGC 4/1/2	34/19/45
4/7	GACAGCTG 14/9/19	CCCAAAATAGCC 4/1/5	CTATAAATAC 7/6/13	N	CCATACAAGGCC 4/1/3	34/17/40
2/7 W	GACAGCTG 14/6/43	TGCCCT 4/1/15	N	GACAGCTGAG 4/1/15	ACAAGGCC 4/1/31	34/9/104
3/7 W	ACAGCTGC 14/8/21	AGGGCA 4/1/12	GGGCTATAAA 7/2/9	AGGGCAGC 4/1/37	N	34/12/79
4/7 W	CAGCTGTT 14/9/15	CCAAATATGG 4/2/3	CCTAAGAATAGC 7/2/5	N	CATACAAGGC 4/1/2	34/14/25
Compare-Prospector	CTGTSA 14/1/4	KAGCYATA 4/1/1	GYTATW 7/5/7	CAGCTGTS 4/1/4	N	34/8/16
Toucan ^7^	GGGrmAGG 14/1/5	N	N	N	CCTGCT 4/2/12	34/3/17

^1 ^x/y/z in the table denotes: experimentally determined binding sites/overlap between experimental sites and predicted sites/predicted sites by a discovered motif.

^2 ^Refer to Figure 1 for the description of '*t*/7' as well as '*t*/7 W'.

^3 ^'N' in a table cell indicates that the corresponding motif was not detected.

^4 ^Representation of degenerated nucleotides: M = (AC), S = (GC), V = (AGC), R = (AG), Y = (CT), H = (ACT), W = (AT), K = (GT), D = (AGT), B = (GCT), N = (AGCT)

^5 ^None of the motifs reported by our approach, CompareProspector or Toucan predicted the experimentally determined Sp1 binding site.

^6 ^Masking repeats represents the 5000-bp upstream sequences (no gap), for which only the repeat regions were masked, were used.

^7 ^Motifs identified by Toucan were taken from their report [7].
